# Supplementary material for: The causal relationship between the human gut microbiota and pyogenic arthritis: a Mendelian randomization study
Source: Front Cell Infect Microbiol. 2024 Nov 26;14:1452480. doi: 10.3389/fcimb.2024.1452480 (PMC11629706; doi:10.3389/fcimb.2024.1452480)
Supplement: Supplementary file 1 [file DataSheet1.zip › mendelian test/ebi-a-GCST90027653.csv_forest.pdf]

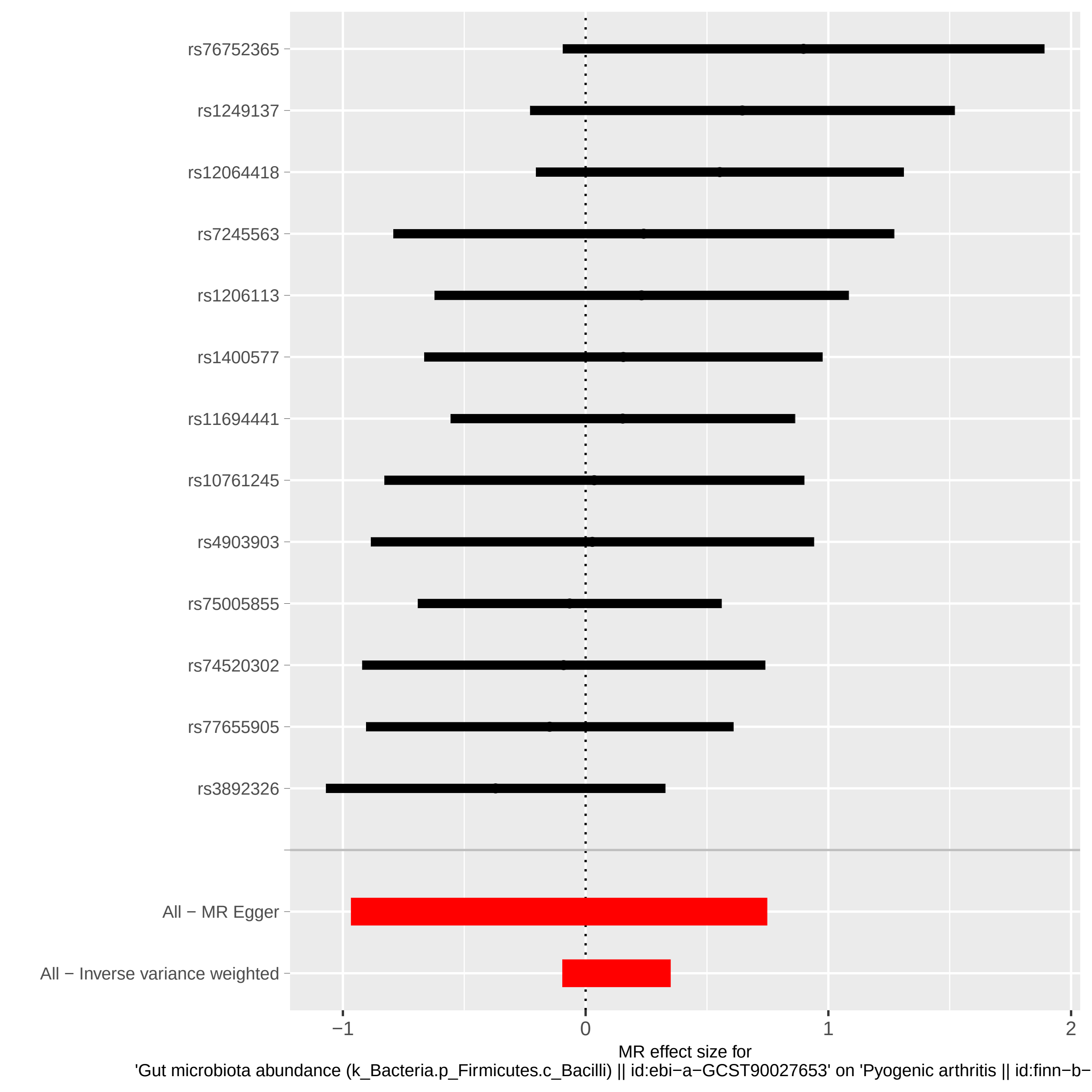
rs76752365
rs1249137
rs12064418
rs7245563
rs1206113
rs1400577
rs11694441
rs10761245
rs4903903
rs75005855
rs74520302
rs77655905
rs3892326
All − MR Egger
All − Inverse variance weighted
−1
0
1
2
MR effect size for
'Gut microbiota abundance (k_Bacteria.p_Firmicutes.c_Bacilli) || id:ebi−a−GCST90027653' on 'Pyogenic arthritis || id:finn−b−
